# Efficacy of the finite element analysis in assessing the effects of light curing on the mechanical properties of direct restorative composites: A systematic review

**DOI:** 10.4317/jced.62021

**Published:** 2024-11-01

**Authors:** Eliana Pineda-Vélez, Pradeep Kumar Yadalam, Carlos M. Ardila

**Affiliations:** 1 Endodontist. Master in Epidemiology. Associate Professor. Institución Universitaria Visión de Las Américas, Medellín Colombia; 2 MDS. Department of Periodontics, Saveetha Dental College, and Hospitals, Saveetha Institute of Medical and Technical Sciences, Saveetha University, India; 3 Ph.D. Postdoc. Titular Professor. Biomedical Stomatology Research Group, School of Dentistry. Universidad de Antioquia U de A, Medellín Colombia

## Abstract

**Background:**

Previous studies have identified the effects of light curing techniques on both shrinkage strain and contraction stress buildup in composite restorations. Finite Element Analysis (FEA) has several advantages over other experimental methods for evaluating the mechanical properties of direct dental resins. The objective of this systematic review is to assess the impact of light curing protocols on the shrinkage behaviors and other mechanical properties of direct restorative composites utilizing FEA.

**Material and Methods:**

The search methodology adhered to the PRISMA guidelines and utilized prominent scientific databases. This systematic review was structured around a question formulated PICO framework. To estimate the methodological rigor of the included studies, a quality assessment tool was utilized.

**Results:**

After the final phase of eligibility evaluation, the systematic review incorporated nine studies. Studies employing FEA primarily aimed to investigate the effects of various light curing protocols on shrinkage behaviors, contraction stress, and microleakage in composite restorations. Most FEA models in these studies incorporated key time-dependent parameters related to composite polymerization, such as shrinkage, Young’s modulus, Poisson ratio, and resulting creep. FEA can provide valuable insights into the effects of light curing on the mechanical properties of direct restorative composites, its accuracy, and reliability depend on various factors, including the accuracy of input parameters, modeling assumptions, and validation against experimental data.

**Conclusions:**

The findings underscore the importance of considering various factors such as curing protocol, testing method, composite characteristics, and environmental conditions in understanding, and mitigating the adverse effects of polymerization shrinkage in composite restorations.

** Key words:**Finite Element Analyses, Composite Resins, Light Curing of Dental Resins, Polymerization, Materials Testing, Mechanical Tests.

## Introduction

Resin composite, the primary material employed for direct dental restorations, facilitates the application of additive restorative techniques and minimally invasive procedures (1). Essential inquiries regarding the clinical efficacy of composite restorations and their potential failures remain pertinent to oral health care. In a recent investigation involving more than 3.5 million composite restorations, it was found that approximately 59% of the restorations survived for 5 years, 43% survived for 10 years, 34% for up to 15 years, and 7% of the teeth that had been restored with resin composite were extracted after 15 years (2).

Material and technological factors influence the clinical effectiveness of resin composite restorations. For example, the composition of the composite resin, including the type and size of filler particles, can affect its mechanical properties, wear resistance, and aesthetics (3). The ability of the composite resin to match the natural color of the tooth is crucial for esthetics and patient satisfaction (4). The type of adhesive system used to bond the composite resin to the tooth structure can affect the longevity and retention of the restoration (5). The handling properties of the composite resin, such as flowability, viscosity, and ease of manipulation, can affect the placement and adaptation of the restoration. Moreover, the curing mechanism of the composite resin can influence the depth of cure, degree of polymerization, and bond strength to the tooth structure (6). *In vitro* experiments have been conducted to test the physical mechanical properties of resin composites, including flexural strength, elastic modulus, microhardness, and compressive strength. Laboratory studies that observe differences between restorative materials and techniques frequently conclude that these discrepancies may be related to the clinical performance of restorations. Examples include varied cavity preparation designs, inactivation of dentin metalloproteinases during bonding phases, gradual filling approaches, and light polymerization strategies (7).

Previous studies (8,9) have identified the effects of light curing techniques on both shrinkage strain and contraction stress buildup in composite restorations. These effects have been linked to the occurrence of immediate or postoperative restorative failure (8,9). It has been noted that all resin-based composites undergo shrinkage during the curing process. This results in stress being generated within the tooth structure (10). As per the American Dental Association, 40% of dentists express apprehension regarding the adverse impacts of polymerization shrinkage. There is evidence to suggest that the inherent stress in a restored tooth could lead to enamel fractures, post-operative dental sensitivity, margin detachment, secondary cavities, and premature restoration failure (10-12). Data supporting this significance is rarely presented or verified in clinical trials (7). However, a systematic study assessed various mechanical properties of resin composites and compared the findings to clinical trial results for the same materials. Interestingly, positive associations between clinical and laboratory outcomes were observed (13).

Finite element analysis (FEA) is a numerical method used to solve complex engineering problems by dividing the problem domain into smaller, simpler elements. Each element is then analyzed to determine its behavior and interactions with neighboring elements (14). FEA has several advantages over other experimental methods for evaluating the mechanical properties of direct dental resins. FEA can predict the mechanical behavior of dental resins under various loading conditions, including tensile, compressive, and shear forces (15). This allows researchers to simulate the performance of dental restorations in different clinical scenarios, such as chewing, biting, and grinding. FEA is a cost-effective method for evaluating the mechanical properties of dental resins, as it does not require expensive laboratory equipment or materials. It also allows researchers to conduct virtual experiments, which can save time and resources compared to traditional experimental methods (16). FEA allows researchers to easily modify the geometry, material properties, and loading conditions of dental resins. This flexibility enables researchers to investigate the effects of different design parameters on the mechanical behavior of dental restorations (17). FEA is a non-destructive method for evaluating the mechanical properties of dental resins. This means that researchers can analyze the behavior of dental restorations without damaging or altering the specimens. FEA can perform sensitivity analysis to determine the effects of small changes in material properties or geometry on the mechanical behavior of dental resins (18). This allows researchers to identify critical parameters that influence the performance of dental restorations. FEA provides visual representations of stress, strain, and displacement distributions in dental resins (19). FEA has proven to be an effective method for examining shrinkage deformations (20) and the quantity and distribution of contraction stresses (21). In instances of polymerization shrinkage, the settings for material properties should consider composite shrinkage strain, related changes in stiffness, and the gradient polymerization at different depths of restoration (22). This allows researchers to easily interpret and communicate the results of their analyses. Overall, FEA is a powerful tool for evaluating the mechanical properties of direct dental resins, as it provides predictive capability, cost-effectiveness, flexibility, non-destructiveness, sensitivity analysis, and visualization capabilities (19-22).

On the other hand, while FEA involves computational methods, it is not inherently artificial intelligence. However, artificial intelligence techniques can be used to enhance FEA, such as in optimization algorithms that can automatically refine mesh elements to improve accuracy or in machine learning algorithms that can predict material properties based on data (23). The findings of a systematic review published in 2018, which evaluated polymerization shrinkage stress through direct examination, indicated that the use of alternative light-curing sources seemed to have a more significant impact in reducing polymerization shrinkage stress compared to alternative material placement techniques or modified photo-activation modes (24). This underscores the importance of further investigating the effects of light curing on the mechanical properties of resin materials using predictive models like FEA.

Considering the importance of FEA in the assessment of the mechanical properties of direct restorative composites, it is important to evaluate the efficacy of this numerical model in assessing the effects of light curing protocols. The use of FEA in this systematic review allows for the prediction and evaluation of the influence of various light-curing protocols on the mechanical properties of direct restorative composites. By simulating the curing process and its effects on the material at a microstructural level, FEA enables a comprehensive understanding of the mechanical behavior of the composite under different curing conditions. This predictive capability is crucial for optimizing the light curing protocols to enhance the mechanical performance and longevity of direct restorative composites. However, there is no known systematic review that has performed such an evaluation. Therefore, the objective of this systematic review is to assess the impact of light curing protocols on the shrinkage behaviors and other mechanical properties of direct restorative composites utilizing finite element analysis.

## Material and Methods

-Protocol and enrollment

The search strategy employed in this systematic review adhered to the recommendations set forth by PRISMA (Preferred Reporting Items for Systematic Reviews and Meta-analyses) (25). The systematic review protocol was officially registered on the Open Science Forum Database and can be accessed using the identifier: osf.io/ny95g

-Criteria for Eligibility

This systematic review was structured around a question formulated utilizing the Population, Intervention, Comparison, and Outcomes (PICO) framework:

P: Studies investigating the application of Finite Element Analysis 

I: Use of light-curing in direct restorative composites

C: Comparative control experiments

O: Evaluation of shrinkage behaviors and other mechanical properties

Studies investigating the application of finite element analysis to evaluate shrinkage behavior and other mechanical properties of light-cured direct restorative composites were included.

Excluded from consideration were conferences, editorials, abstracts, systematic and narrative reviews, meta-analyses, and investigations lacking essential details regarding methodologies.

-Sources of Information

The search strategy included accessing renowned scientific databases like PubMed/MEDLINE, EMBASE, SCOPUS, and SCIELO, as well as exploring grey literature sources. A comprehensive electronic database search covered material from the inception of these databases up to June 2024, with no language limitations. Supplementary records were found by reviewing the reference lists and citations within all chosen full-text documents for potential inclusion in the systematic review.

-Search Approach

The search strategy entailed utilizing the following search terms: “Finite Element Analysis,” OR “FEA,” OR “Finite Element Model” OR “FEM” AND “light-curing,” AND “direct composite resins,” AND “shrinkage behaviors” AND “contraction stress” AND “microleakage” AND “restorative dentistry,” AND “dentistry.” This method was initially devised for PubMed and subsequently adjusted for other databases.

Two authors independently reviewed titles and abstracts for inclusion, followed by a thorough examination of full-text articles. Eligibility based on full-text assessment was determined independently and in duplicate. Any disparities were resolved through discussion. The interobserver agreement was evaluated using the Kappa statistical test, with a threshold of >85 considered statistically significant.

-Data Gathering

Two authors autonomously gathered data utilizing adaptable data extraction techniques. A comparative analysis was conducted on the collected data to ensure coherence. The data comprised information on the application of FEA, including essential details of the predictive model and noteworthy research outcomes. Furthermore, the names of authors and publication years were recorded.

-Assessment of Bias Risk and Study Quality

To estimate the methodological rigor of the included studies, the 16-item Quality Assessment Tool for Studies with Diverse Designs (QATSDD) was utilized (26). This tool comprises 16 criteria, addressing elements such as a clear theoretical framework, stated objectives, detailed research setting description, justification of sample size adequacy, the inclusion of a representative sample, thorough explanation of data collection procedures, rationale behind chosen data collection tools, comprehensive recruitment details, statistical assessment of tool reliability and validity, alignment between research question and data collection method, coherence between research question and data collection format and content, consistency between research question and analysis method, well-founded justification for analytical approach, evaluation of analytical process reliability, demonstration of user involvement in the design, and critical discussion of strengths and limitations. Each criterion holds equal weight and is rated on a scale from 0 to 3 (0 = insufficient detail, 1 = inadequately provided, 2 = moderately provided, 3 = comprehensively provided). The cumulative score across these criteria yields an overall assessment of the evidence, expressed as a proportion of the maximum achievable score.

-Summary Statistics

Descriptive statistics, including mean differences and standard deviations, were employed to extract data from the studies included, particularly for continuous outcomes. If the included papers demonstrated a certain level of similarity, the feasibility of conducting a meta-analysis was considered as a potential follow-up.

Ethical approval was not required for this study.

## Results

-Study Identification

**Figure 1:**
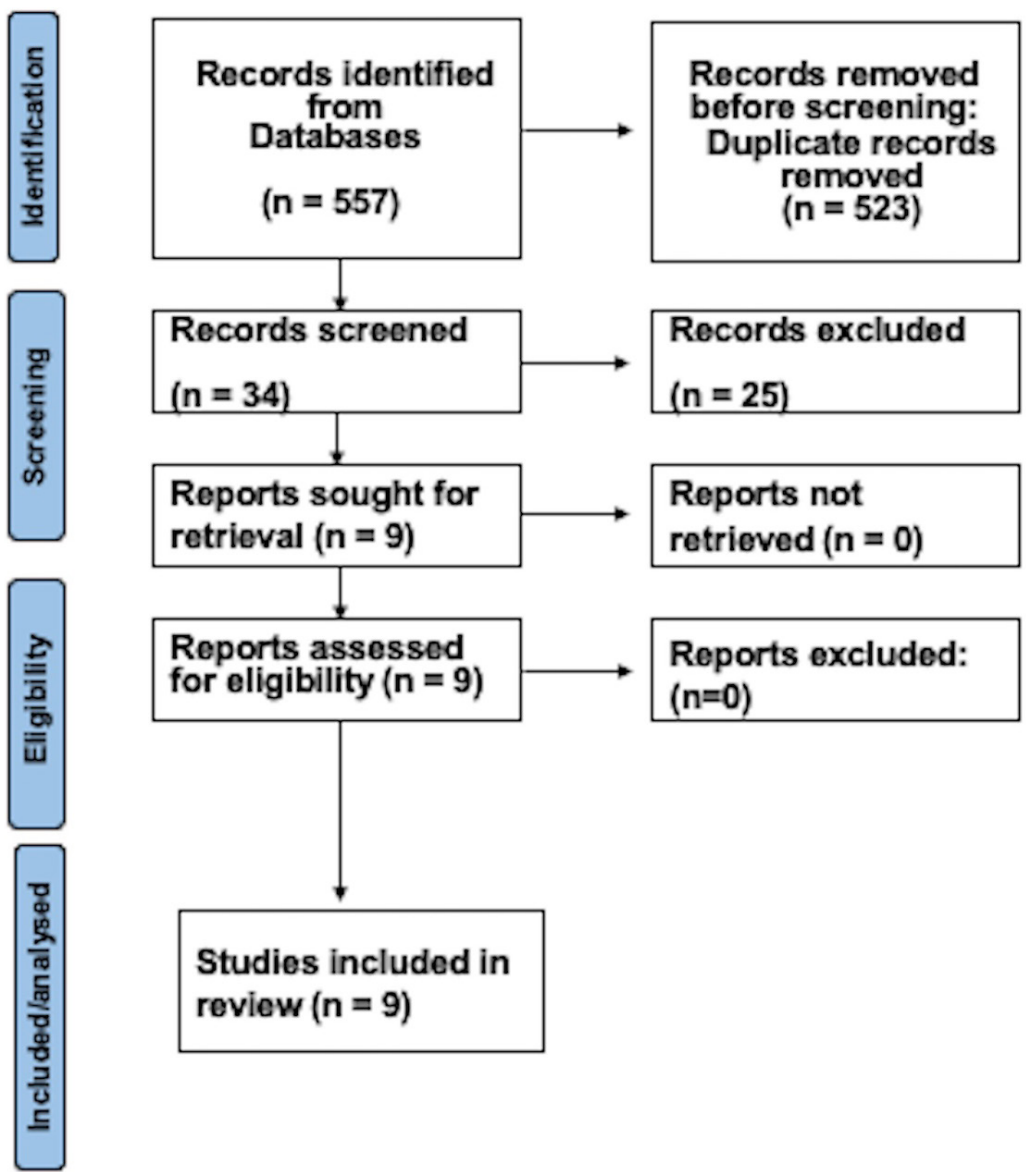
Schema of the selection method.

After executing the search strategy, 557 studies were initially located in electronic databases. Subsequent removal of duplicate entries and application of eligibility criteria led to 34 articles being subjected to a thorough full-text assessment. During this review phase, studies were primarily excluded due to insufficient emphasis on light-curing outcomes. Following the conclusive evaluation of eligibility criteria, the systematic review encompassed nine papers. Figure 1 provides a comprehensive depiction of the search process.

-Characteristics of the Studies

 Table 1 summarizes the descriptive features of the nine studies incorporated in this systematic review (22,27-34). The publications analyzed in this review range from 2000 (34) to 2016 (22). Most of the studies were conducted in North America and Europe. Studies employing FEA primarily aimed to investigate the effects of various light curing protocols on shrinkage behaviors, contraction stress, and microleakage in composite restorations.

-Primary Results

 Table 2 illustrates the characteristics of the FEA utilized in each study. All finite element models effectively fulfilled the objectives outlined by their respective investigations and exhibited the validity, accuracy, and reproducibility of the FEA. Most FEA models in these studies incorporated key time-dependent parameters related to composite polymerization, such as shrinkage, Young’s modulus, Poisson ratio, and resulting creep. These parameters were adjusted during simulations using custom software subroutines, ensuring that variations occurring in shrinkage, Young’s modulus, Poisson ratio, and creep during composite polymerization were accurately represented in the models.

The FEAs used in this review encompassed various methodologies and applications.

Analysis of Restorations: The strains from unbonded restorations were integrated into two FEA models, each defining the composite as either homogeneous or exhibiting polymerization gradients. Initial solutions were validated through deformations in bonded restorations. Micro-CT scanning assessed interfacial microleakage, with comparisons made to FEA results (22).

Simulation of Shrinkage: Dome and disk-shaped samples underwent shrinkage simulation using FEA with axisymmetric elements. Dimensional changes were accounted for in three dimensions, incorporating each directional shrinkage component (27).

Construction of Geometric Models: A 3D geometric model was constructed from 33 cross-sections of tooth 25, meshed using the FEA method. Models integrated components like the maxillary premolar, pulp cavity, and composite restoration for various cavity configurations, simulating polymerization shrinkage forces (28).

Investigation of cavity-factor (C-factor) impact: Six FEA models were developed to study the effect of C-factor on interfacial shrinkage stress, utilizing a contact pair to simulate the interface between tooth structure and composite resin (29).

Integration of Custom Sub-routine: A custom sub-routine was incorporated into the FEA software to integrate the model numerically, using parameters from previous experiments to simulate reaction kinetics and polymerization shrinkage. Simulation comparisons were made with experimental data (30).

Time-dependent FEA Modeling: Time-dependent FEA modeling utilized 2D axisymmetric geometries, ensuring convergence and comparison with *in vitro* experiments to validate the model (31).

Micro-CT Imaging: Micro-CT imaging replicated internal dental structures for interface stress computation, incorporating time-dependent parameters of composite polymerization (32).

Dynamic Mechanical Analysis: A quadrant model was created to determine optimal preload and amplitude for loading a composite specimen within a PTFE tube, utilizing FEA analysis alongside a dynamic mechanical analyzer (33).

Quasi-static Process Modeling: An axisymmetric FEA model was constructed, assuming ideal interface bonds and simulating polymerization as a quasi-static process with discrete depth-of-cure steps (34).

These diverse approaches offer comprehensive insights into the effects of light curing protocols on composite restorations’ mechanical properties.

The results of these studies mainly highlight how different curing protocols affect shrinkage, stress, and microleakage in composite restorations. Vertical curing at standard intensity resulted in the highest levels of these factors (22). Furthermore, optical methods show higher shrinkage values compared to dilatometer methods, with significant differences noted (27). Additionally, adhesive layer stress increases with cavity conFiguration complexity and decreases adhesive bond strength at low temperatures (28). Moreover, a higher conFiguration of the C-factors correlates with increased shrinkage stress and interfacial debonding likelihood (29). Furthermore, a comprehensive model captures the complex relationship between stiffness, flowability, and curing parameters during the polymerization process (30-32). Additionally, chemically cured composites exhibit lower stress during polymerization shrinkage, while hybrid composites demonstrate increased stiffness during light-activated polymerization compared to conventional composites (33). Lastly, FEA simulations highlight the significant influence of flow on polymerization shrinkage (34).

-Results Synthesis

Synthesizing the results, the systematic review opted against a meta-analysis due to notable disparities in methodological approaches and research designs among the included studies. While various FEA designs were examined, several key references for outcome evaluation and consideration of different mechanisms were identified. Consequently, the analysis was limited to a qualitative assessment.

-Bias Risk and Study Quality in Individual Studies

Assessment of bias risk and study quality revealed that all studies met at least 75% of the established quality criteria (26), categorizing them as of good quality (Table 3). Although most studies lacked sample size calculation and representation of a diverse sample, it’s noteworthy that they fulfilled the remaining criteria assessed by the evaluation method.

## Discussion

This is the first systematic review that evaluates the efficacy of the finite element model in assessing the effects of light curing on the mechanical properties of direct restorative composites.

FEA serves as a pivotal tool in biomechanical assessments of adhesive bonds within composite restorations. It embodies a sophisticated, contemporary approach known for its exceptional reproducibility and non-invasive nature, enabling in-depth exploration of the biomechanical dynamics of restored dental structures (19-22,28). FEA offers a means to scrutinize stress distributions post-light-curing of composite restorations and the effects of temperature fluctuations, effectively simulating oral cavity conditions and serving as a predictive and evaluative instrument (14-17,22,28-31). Through meticulous manipulation of mathematical models—via segmentation, magnification, and rotation—a comprehensive understanding emerges regarding load transmission and dispersion across interfaces of hard dental tissues and adhesive-composite materials, typically obscured in clinical settings (18-22,28,32-34). Its foremost advantage lies in elucidating the combined influence of interdependent factors replicating clinical scenarios: mechanical properties of restorative materials, cavity morphology, and interface integrity (adhesive bonding quality) (22,27-34). Notably, FEA excels in its ability to visualize variables in three-dimensional space, offering color-coded representations and dynamic simulations to simplify the interpretation of intricate mathematical data, thereby enhancing comprehension and decision-making processes (14-22,27-34).

Previous studies have acknowledged the impact of light curing protocols on the shrinkage strain and the development of contraction stress in composite restorations (8,9,22). This influence has been associated with immediate or postoperative restorative failures (9,22). Several methodologies have been employed to evaluate shrinkage behaviors and their temporal variations concerning polymerization kinetics, with the bonded disc method being commonly utilized in various investigations (35,36). An analogous experimental setup has been devised to map shrinkage profiles on flat composite materials utilizing a stylus-dial gauge system (37). Nonetheless, the shrinkage dynamics within dental cavities pose a more intricate challenge compared to measurements taken outside cavities. Studying stress-strain kinetics becomes arduous without measurements furnishing spatially resolved information (22). Another prevalent technique for evaluating interfacial debonding between the tooth and composite resin is dye penetration. This method involves immersing the sample in a dye solution, sectioning it into slices, and examining it under a profilometer or microscope (38). While the dye penetration approach is widely used, its procedure is both damaging and labor-intensive, and the insights gained from 2D sectional views are restricted. As a result, non-destructive 3D technologies such as X-ray microcomputed tomography have been used to evaluate the interfacial state of composite restorations (39). However, due to its limited resolution, X-ray microcomputed tomography cannot identify debonding at the submicron level. Furthermore, none of the approaches can monitor debonding in real time; instead, they can only analyze the restoration process after polymerization (29).

Conversely, conducting a FEA simulation necessitates the modeling of geometries, materials, and loading/boundary conditions. Regarding polymerization shrinkage, the material properties settings should encompass the shrinkage strain of composites, along with their corresponding stiffness alterations and gradient polymerization at various depths of the restorations (22). The primary challenge in studying shrinkage behavior stems from the variability of the polymerization process under different light-curing conditions, further compounded by the intricate anatomical conFiguration of composite restorations (22,29). Moreover, the surrounding tooth partially shields light irradiation, although enamel and dentin exhibit varying degrees of light permeability (22). As noted in this systematic review, most studies utilize assumptions to streamline the settings and facilitate the resolution process. However, additional technologies have been introduced to complement FEA and enhance its outcomes. Examples comprise the digital image correlation method and acoustic emission.

The digital image correlation method was employed to examine the contraction behavior of dental composites in both free shrinkage and restoration forms. By comparing two images captured at different times, displacements and gradients of specific points can be calculated using pattern-matching assumptions on defined subjects (22,40). The measures assisted by digital image correlation aid in evaluating both the temporal and spatial dependence of shrinkage strain (22), as well as the global contraction fields of composite restorations (22,40). Furthermore, their findings can be utilized to validate simulations using numerical analysis, which investigates contraction stress (38-40). As evidenced by this systematic investigation, integrating digital image correlation and FEA into an inquiry model proved to be an effective approach for examining the shrinkage-stress conditions of composite restorations. Indeed, Chuang *et al*. (22) found that both light intensity and direction influence shrinkage and contraction stress. The promising results of the validation procedure demonstrated that this hybrid experimental-numerical analysis method can examine shrinkage behaviors, thereby enhancing the reliability of stress analysis.

The acoustic emission technique utilizes transducers or sensors to detect high-frequency sound waves generated when strain energy is released within a material during fracture. It is a real-time, non-destructive, and highly sensitive method for assessing structural integrity. This technique has been widely employed in both research and industry to monitor the advancement of crack propagation and fracture behavior in diverse structures (29). Hence, the investigation conducted by Liu *et al*. (29), examined in this systematic review, demonstrated the efficacy of acoustic emission and FEA in detecting interfacial debonding of restorations during composite resin polymerization.

As evidenced in this systematic review, several studies have employed FEA to investigate the biomechanics of polymerization shrinkage, the evolution of shrinkage stress, and their impact on restorative quality. Due to the multifactorial nature of boundary conditions and shrinkage behaviors, these simulations are intricate and necessitate validation (22,29). For instance, a study referenced in this review demonstrated that FEA models provided an accurate deformation profile of free surfaces compared to displacements on bonded restoration boundaries (22). However, disparities were observed in displacements on bottom surfaces, where actual measurements surpassed analytical results. It is speculated that interfacial debonding occurred during polymerization, allowing the composite to shrink more freely. The micro-CT analysis unveiled notably higher levels of microleakage at the bottom region. Conversely, in the FEA simulations, the composites were assumed to be fully adhered to the tooth, constraining their deformations (22,29).

Overall, while FEA can provide valuable insights into the effects of light curing on the mechanical properties of direct restorative composites, its accuracy, and reliability depend on various factors, including the accuracy of input parameters, modeling assumptions, and validation against experimental data (22-24). FEA often relies on simplifications and assumptions in modeling the complex behavior of materials and their interaction with light curing (23-26). These simplifications may not fully capture the intricate details of the curing process and its effects on mechanical properties. Moreover, FEA requires accurate input parameters, including material properties such as modulus of elasticity, Poisson’s ratio, and shrinkage strain (27-30). However, obtaining precise material properties for restorative composites, especially under varying curing conditions, can be challenging (31-34). As observed in the studies reviewed here, validating FEA predictions against experimental data is crucial for ensuring the accuracy and reliability of the analysis. Another limitation identified in this systematic review stemmed from the challenge of conducting quantitative analysis. This limitation largely stemmed from methodological and design discrepancies observed across the reviewed studies. These studies encompassed diverse research designs, utilized multiple essential references for outcome assessment, and explored various FEA methodologies with varying designs.

## Conclusions

FEA confirms that different curing protocols lead to varying levels of shrinkage strain, contraction stress, and microleakage. Vertical curing at standard intensity results in the highest levels of these parameters, while lower intensity and oblique curing mitigate some effects but do not eliminate them entirely. FEA reveals significant stress concentration within the adhesive layer at low temperatures, where forces during polymerization may exceed adhesive bond strength. Environmental conditions, such as temperature, greatly influence mechanical behavior. Stress progression is influenced more by composite characteristics like stiffness, flowability, and curing rate than by the level of shrinkage exhibited. This highlights the importance of selecting composites with desirable mechanical properties. FEA shows variations between composite types in terms of mechanical behavior during polymerization. While the degree of conversion remains consistent, storage modulus differs, with hybrid composites exhibiting notably higher values.

In summary, the findings underscore the importance of considering various factors such as curing protocol, testing method, composite characteristics, and environmental conditions in understanding, and mitigating the adverse effects of polymerization shrinkage in composite restorations.

## Figures and Tables

**Table 1:** Descriptive characteristics of the studies included.

Authors and publication year	Country	Main aim
Chuang et al. 2016 (22)	Taiwan	To examine how different light curing protocols impact the shrinkage behaviors, contraction stress, and microleakage in composite restorations.
Tantbirojn et al. 2015 (27)	USA	A basic optical technique for assessing polymerization shrinkage of dental composites is contrasted with a dilatometer method.
Manchorova-Veleva 2011 (28)	Bulgaria	To investigate and assess the magnitude of stresses within the adhesive bond in composite restorations of masticatory teeth following light-curing, amidst temperature variations and masticatory forces.
Liu et al. 2011 (29)	China	This study explored the impact of the C-factor on interfacial debonding during the curing process of composite restorations, employing the acoustic emission technique.
Koplin et al. 2009 (30)	Germany	To contrast the accumulation of internal stresses in four distinct dental composites during the curing process, drawing on findings from a prior study on polymerization kinetics, and to delineate the evolving mechanical properties for various activation methods.
Jakubinek et al. 2008 (31)	Canada	To construct and assess a model for simulating temperature rises during the light-curing process of dental restorations, and to employ this model to examine the impact of various factors on the peak temperature increase along the pulp-dentin junction.
Kuijs et al. 2003 (32)	Netherlands	To contrast the shrinkage stresses among various restorative methods employed for cusp-replacement restorations using direct resin composite.
Sakaguchi et al. 2002 (33)	USA	To assess the feasibility of employing dynamic mechanical analysis on tubular geometry within a three-point flexure fixture to monitor the development of storage modulus in a light-activated polymer matrix composite.
Winkler et al. 2000 (34)	USA	To authenticate a Finite Element Method approach for examining polymerization shrinkage.

**Table 2:** Main findings.

Authors	Finite Element Analysis characteristics and operation	Effects of light curing	Main results
Chuang et al. 2016 (22)	The strains observed in the unbonded restorations were incorporated into two finite element analysis models, where the composite was defined either as homogeneous or exhibiting polymerization gradients. Initial solutions were validated through their respective deformations in the bonded restorations. Additionally, micro-CT scanning was utilized to assess the interfacial microleakage of the restorations, with comparisons drawn between the results obtained from FEA simulations.	Three sets of human molars were gathered to undergo varied light-curing procedures: vertical or oblique curing at standard intensity, and vertical curing at diminished intensity. Each tooth received composite fillings successively under both unbonded and bonded conditions, and their shrinkage behaviors were assessed using digital image correlation methodology.	Vertical curing at standard intensity resulted in the highest levels of shrinkage strain, contraction stress, and microleakage among the three protocols. Lower intensity curing reduced overall shrinkage strain and displacements at the cervical margin but did not eliminate microleakage formation. Oblique curing induced asymmetric shrinkage, with less deformation observed on the side shielded by the tooth.
Tantbirojn et al. 2015 (27)	The shrinkage of both dome and rounded disk-shaped samples, as described in the experimental methods, was simulated using finite element analysis with four-node, isoparametric, arbitrary quadrilateral axisymmetric elements. In FEA, dimensional changes are accounted for in three dimensions. Consequently, each directional shrinkage component is included in linear shrinkage terms during the simulation.	As the materials underwent light-curing through the glass slides (for 40 seconds), mercury levels were monitored and recorded over 60 minutes (N = 6) to assess volumetric shrinkage. Meanwhile, for the optical method, uncured composites were placed on a smooth silicone platform. A pre-polymerization image was captured under a stereomicroscope before light-curing (40 seconds). Subsequent post-polymerization images were taken at intervals of 2, 10, 60, and 90 minutes (N = 10).	At the 60-minute mark, volumetric shrinkage ranged from 1.24% to 2.24% for the dilatometer method and 1.35% to 2.68% for the optical method. The shrinkage values obtained through the optical method consistently exceeded those from the dilatometer (P = 0.0001), though the ranking of the composites remained consistent. FEA indicated that the lower shrinkage values observed with the dilatometer method could be attributed to the bonding of its samples.
Manchorova-Veleva 2011 (28)	A total of 33 cross-sections from tooth 25 were chosen and utilized in constructing a 3D geometric model, which was meshed using the finite element method. Each model incorporated the maxillary premolar, pulp cavity, periodontal ligament, adhesive layer, and composite restoration generated through finite element analysis. These components were integrated into a unified model for each of the eight cavity configurations. A comparative computer simulation was conducted to assess polymerization shrinkage forces of the composite material.	Analysis of stresses in the adhesive bond within composite restorations of masticatory teeth was conducted following light-curing, under varying temperature conditions, and during masticatory loads. The stress distribution on the adhesive bond was examined across eight distinct class I and II cavity configurations. Furthermore, the occurrence of crack formation was evaluated in instances of adhesive bond rupture.	In each cavity configuration, there's a notable increase in stress concentration within the adhesive layer at the interface with dental tissues. At low temperatures (5°C), the forces generated exceed the adhesive bond strength across all cavity configurations studied. This exacerbates the negative impacts of polymerization shrinkage in composite restorations of masticatory teeth. Additionally, the distribution of generated stresses under axial and tangent forces of 300 N mirrors that under the influence of temperature factors.
Liu et al. 2011 (29)	Six Finite Element Analysis models were constructed to investigate the impact of the C-factor (configuration of the cavity) on interfacial shrinkage stress. The interfacial bond between the tooth structure and the composite resin was simulated using a contact pair, which linked the two surfaces together. This approach enabled the evaluation of stresses both perpendicular and parallel to the interface.	The cavities were treated with adhesive and filled with composite, which underwent curing with a halogen light for 40 seconds. An acoustic emission (AE) system with 2 channels was employed to track interfacial debonding, resulting from shrinkage stress, between the tooth and the restoration. An AE sensor was affixed to the specimen's surface for this purpose. AE recording commenced concurrently with composite curing and continued for 10 minutes. A thermal load, involving a decrease in temperature, was imposed on the composite resin to mimic its shrinkage.	Based on the findings from both the AE tests and FEA simulations, it can be inferred that as the C-factor increases, so does the magnitude of shrinkage stress, consequently elevating the likelihood of interfacial debonding.
Koplin et al. 2009 (30)	A custom sub-routine was incorporated into the finite element software to numerically integrate the model. The parameters describing reaction kinetics and polymerization shrinkage were derived from previous experiments. To assess the consistency of the parameter set representing the mechanical properties of the curing composite, a 3D finite element simulation of the experiment was conducted and compared with experimental data. Following this, 3D finite element simulations were performed for various filling geometries and activation modes.	A halogen light source with an output of 400 mW/cm2 was utilized for photo-activation. To modulate the light intensity, a neutral optical filter was employed. Measurements were performed using this optical filter to adjust the light intensity. The reduced light intensity was compensated for by prolonging the exposure time, ensuring a constant total energy input. Two photo-activation modes were examined: a 40-second exposure at 100% transmission and an 80-second exposure at 50% transmission.	Employing a model that captures the intricate relationship among stiffness, flowability, curing rate, and activation intensity during the curing process provides a more comprehensive understanding of the spatial and temporal accumulation of stresses. A favorable reaction kinetics or reduced stiffness can offset the impact of increased polymerization shrinkage on the peak stress observed. The progression of stress is not directly correlated with the level of shrinkage exhibited by the composites.
Jakubinek et al. 2008 (31)	Time-dependent FEA modeling was conducted using 2D axisymmetric geometries. The results were scrutinized for convergence, ensuring that there was no notable reliance on the number of elements or time-step size. The data obtained from the FEA models were juxtaposed with in vitro experiments. To authenticate the FEA model, the temperature elevation recorded by the thermocouple was juxtaposed with the temperature rise near the pulp horn in the model.	To derive meaningful outcomes from FEA simulations concerning the temperature rise at the pulp-dentin junction, it was essential to establish standard properties for light-cured dental composites. The necessary material properties including heat capacity, density, enthalpy of polymerization, thermal conductivity, reflectance, and light penetration depth were determined through a combination of literature review and experimental investigations.	Comparisons with laboratory experiments suggest that the model offers a reliable estimate of real temperature elevations. Key factors influencing this include the intensity and duration of the curing light, as well as the enthalpy of polymerization of the resin composite. The composite material exhibits notable insulating properties, with the highest risk occurring during light curing of thin layers of bonding resin or in deep restorations lacking a liner to serve as a thermal barrier.
Kuijs et al. 2003 (32)	Micro-CT imaging was employed to replicate the internal structure of dentin, pulp, enamel, and a cusp-replacing restoration within an upper premolar. Interface stresses were computed based on the internal nodal forces at the interface. The model encompassed the primary time-dependent parameters of composite polymerization.	The model simulated two restorative methods. The chemically cured composite in bulk was modeled with a setting time of 15 minutes. However, for the bulk light-cured composite, this duration was rescaled to 30 seconds, thereby necessitating the rescaling of all relevant parameters. In the case of other procedures involving layering, each layer was modeled with a setting time of 30 seconds, with a one-minute interval between layers. Following the setting of the final layer, the stresses were computed.	When accounting for polymerization shrinkage, it was observed that a chemically cured composite exhibited the least resulting stress. The variations observed among different layering build-up techniques were not as pronounced as anticipated. The findings suggest that the stress-bearing areas are primarily the interface and the cervical part of the remaining cusp.
Sakaguchi et al. 2002 (33)	An FEA model of the test setup was developed to establish an optimal preload and amplitude for allowing the loading knife to deform the composite specimen within the PTFE tube. A quadrant model was constructed using quadrilateral brick elements. A dynamic mechanical analyzer, operating in stress control mode, applied a sinusoidal deflection at 200 Hz. The FEA analysis indicated that a preload of 0.1 N and a deflection of 20 μm would adequately deform the outer PTFE sheath to facilitate load transfer to the inner composite core.	The contact analysis presumed that the composite had no elastic modulus at the beginning of the experiment and adopted linear elastic material properties five minutes after light activation. Two curing light guides, each with an outer diameter of 12.6 mm, were positioned at a 45-degree angle to the sample. The distance between the light guide and the sample was 10 mm. Following an initial equilibration period of 30 seconds, the samples were cured for 1 minute.	This approach assesses the increase in storage modulus during light-activated polymerization. While the degree of conversion did not exhibit significant differences at 1 hour compared to 13.5 minutes after light activation for both composites, the hybrid composite demonstrated a notably higher storage modulus at 1 hour (P=0.03).
Winkler et al. 2000 (34)	An axisymmetric was constructed. Consistent with prior studies, ideal interface bonds were assumed. Polymerization was modeled as a quasi-static process, with the continuously increasing depth-of-cure simulated through a series of eight discrete steps. At each step, every additional increment of composite was considered fully cured.	To ensure that the freely movable components could achieve stable positions and facilitate a steady baseline measurement, the displacement of the profilometer tip was electronically recorded for 1 minute immediately before activating the resin with the light. Subsequently, the resin was polymerized using a hand-held curing light positioned approximately 8 cm above the surface, with a cylindrical jig serving as a reference distance.	The FEA methodology enables simulation of the polymerization process, revealing that the influence of flow on effective polymerization shrinkage could be significant.

**Table 3:** Bias Risk and Study Quality in Individual Studies (26).

Study	Criteria completely satisfied	Percentage score of compliance
Chuang et al. 2016 (22)	14	88%
Tantbirojn et al. 2015 (27)	14	88%
Manchorova-Veleva 2011 (28)	14	88%
Liu et al. 2011 (29)	14	88%
Koplin et al. 2009 (30)	14	88%
Jakubinek et al. 2008 (31)	14	88%
Kuijs et al. 2003 (32)	14	88%
Sakaguchi et al. 2002 (33)	14	88%
Winkler et al. 2000 (34)	14	88%

## Data Availability

The datasets used and/or analyzed during the current study are available from the corresponding author.
